# Exploring the feasibility and acceptability of sensory-adapted menus for adolescents with autism spectrum disorder in a school canteen: the AUT-MENU pilot study

**DOI:** 10.3389/fnut.2026.1978351

**Published:** 2026-10-30

**Authors:** Stefania Ruggeri, Francesca Antonazzi, Ileana Croci, Maria Luisa Scalvedi, Benedetta Rocchi, Maria Vittoria Conti, Chiara Breda, Ilaria Zambon, Sara Basilico, Hellas Cena, Emanuele Marconi

**Affiliations:** 1 Research Centre for Food and Nutrition-CREA, Roma, Italy; 2 Predictive and Preventive Medicine Research Unit, Bambino Gesù Children's Hospital, IRCCS, Rome, Italy; 3 Laboratory of Dietetics and Clinical Nutrition, Department of Public Health, Experimental and Forensic Medicine, University of Pavia, Pavia, Italy; 4 Clinical Nutrition Unit, Istituti Clinici Scientifici Maugeri Istituto di Ricovero e Cura a Carattere Scientifico, Pavia, Italy; 5 Italian Institute for Planetary Health, Milan, Italy; 6 Department of Science and Bio-Technology, Università Campus Bio-Medico di Roma, Rome, Italy

**Keywords:** adolescents, autism spectrum disorder, food selectivity, mealtime behavior, school canteen, sensory-adapted menus

## Abstract

**Background:**

Food Selectivity (FS) is highly prevalent among adolescents with Autism Spectrum Disorder (ASD) and may contribute to unbalanced dietary patterns and adverse nutritional outcomes. This pilot study assessed the feasibility and acceptability of sensory-adapted menus nutritionally planned in accordance with Italian dietary recommendations and Mediterranean dietary principles in a school canteen for adolescents with ASD.

**Methods:**

Eleven adolescents with ASD (14–20 years) participated in a two-phase pilot study consisting of an observational phase (T0) followed by a multicomponent intervention (T1) combining sensory-based menu modifications with changes in mealtime organisation. Sociodemographic and clinical characteristics were collected at baseline. Feeding Problems (FP) and adherence to the Mediterranean Diet (MD) were assessed pre- and post-intervention. Food consumption was assessed using the Pictorial Dietary Assessment Tool (PDAT). Analyses were primarily descriptive and exploratory.

**Results:**

Descriptive changes in food acceptance varied by dish type. Consumption was higher at T1 for selected first courses and fish-based dishes whereas fruit acceptance was lower. Descriptive changes in mealtime-related behaviors were also observed, including greater peer interaction and less frequent excessive smartphone use. The observed changes in food acceptance, FS, dietary habits, and mealtime-related behavior represent preliminary exploratory findings and should not be interpreted as evidence of intervention effectiveness.

**Conclusion:**

Larger controlled intervention studies are needed to investigate the potential of school canteen environments to address food selectivity and support mealtime-related social behaviors among children and adolescents with ASD.

**Clinical trial registration:**

https://clinicaltrials.gov/study/NCT06266377, identifier NCT06266377.

## 1 Introduction

Feeding problems including Food Selectivity (FS), food refusal, neophobia, ritualistic eating behaviors, and sensory sensitivity to some food characteristics, e.g., texture, smell, and color, are very common among children and adolescents with Autism Spectrum Disorder (ASD) (1–5). Individuals with ASD often show marked preferences for soft or semi-liquid textures, for food with low intensity colors, and mild tastes, while they tend to reject strong odors and very hot foods (4, 6). Food selectivity in children and adolescents with ASD is highly heterogeneous, with reported prevalence rates ranging from 21 to 76%, consistently higher with respect to neurotypical individuals (7). Recent evidence has highlighted the role of sensory sensitivity as a potential contributing factor to avoidant/restrictive food intake disorder. Sensitivities to food texture and taste were reported to be approximately 2–10 times more frequent in children with ASD than in children without ASD. However, sensory sensitivities and food preferences vary considerably between individuals with ASD, with other manifestations of food selectivity including difficulties with mixed foods, restricted food preferences, and preferences for specific food brands (7).

At the same time, individuals with ASD may also display a strong preference for ultra-processed foods, which are high in sugar, fat, salt, additives and with a high calorie content (6, 8). The presentation of dishes, along with other sensory stimuli from the mealtime environment, plays an important role, as these factors may cause discomfort in individuals with ASD, negatively affecting their mealtime experience. Some authors have shown that feeding problems are not correlated with the severity of ASD nor with social and communication deficits and other clinical characteristics (9–11); however, the causes of eating difficulties in ASD are not yet fully understood. Compared with neurotypical individuals, FS often persists in adulthood and may lead to clinically relevant eating problems across the lifespan, including micronutrient deficiencies, excessive energy intake and an increased risk of obesity and other comorbidities (12–15).

Over the past decades, intervention studies aimed at reducing FS and improving eating behaviors among children and adolescents with ASD have predominantly been conducted in clinical or home-based settings (6, 10, 16–20) employing heterogeneous methodologies including caregiver-reported questionnaires, self-reported questionnaires, and parent-implemented training programs. In contrast, intervention studies aimed at improving FS and eating behaviors in children and adolescents with ASD in school settings are extremely scarce. A school-based study involving 19 pupils reported significant improvement in FS, food refusal and mealtime behaviors (21); another intervention study was conducted with an 18-year-old girl in a school cafeteria (22). A school-based intervention involving 56 children (8–15 years) with ASD led to an increased acceptance of fruits and vegetables after modification to texture, appearance and temperature (23). However, evidence on sensory-adapted menus integrated into nutritionally balanced, complete school meals under real-world canteen conditions, while also assessing their nutritional composition, remains limited.

To address this gap, the primary aim of the AUT-MENU exploratory pilot study was to assess the feasibility of implementing sensory-adapted, nutritionally balanced menus for adolescents with ASD in a real-world school canteen setting, while also evaluating the nutritional composition of the meals as actually served. Secondary exploratory aims were to describe changes in food acceptance patterns and changes in FS, adherence to the MD and mealtime-related behaviors before and after the intervention.

## 2 Materials and methods

### 2.1 Study design and setting

This pilot feasibility study was conducted within the AUT-MENU project using a single-arm pre-post design without a control group by a multidisciplinary team from the Research Centre for Food and Nutrition (CREA) in Rome and the Laboratory of Dietetics and Clinical Nutrition (LDNC) at the University of Pavia (24). This paper reports data from the pilot study carried out at “Giuseppe Garibaldi-Agricultural Technical Institute” high school in Rome (Lazio Region), from November 2025 to February 2026. Results from the northern Italian study site were previously reported (20).

The AUT-MENU study was registered on ClinicalTrials.gov (registration number: NCT06266377) and the Rome pilot study was approved by the Ethics Committee CET Latium Area 2 at AOU Tor Vergata Polyclinic on 30 September 2025 (project number 126.24).

The present study reports exclusively the school-based Rome component of the broader bicentric AUT-MENU project registered on ClinicalTrials.gov (NCT06266377). Compared with the original registration, the study in Rome underwent some modifications concerning the participating school: participant age range, study timeline, and meal-assessment schedule. The start of the study was postponed due to delays in obtaining ethical approval. Consequently, the upper age limit was extended to 20 years to allow the inclusion of the eligible student who were still attending the school. One of the two schools initially selected for the study could not be included because it was unable to accommodate the required meal modification. The number of meal assessments was reduced from 16 to 12 per participant (six at T0 and six at T1) to accommodate the shortened timeframe and to facilitate participants’ attendance to lunch sessions school. These changes and their underlying reasons are detailed in accordance with the CONSERVE-CONSORT guidelines in the accompanying CONSERVE-CONSORT checklist.

The pilot study was conducted in accordance with the principles of the Helsinki Declaration and was in line with the principles of Good Clinical Practice.

### 2.2 Enrolment and eligibility criteria

In May 2025, CREA researchers contacted the teachers at the participant’s school and two meetings were organized with students with ASD, their families, and school personnel (support staff, teachers and kitchen staff) to present the study, provide detailed information about the inclusion and exclusion criteria, and answer any question.

Inclusion criteria for students were a clinically confirmed diagnosis of ASD according to the Diagnostic and Statistical Manual of Mental Disorders (DSM-5) (25), age 14–20 years, and current enrolment in the participating school. Exclusion criteria were inability to eat independently due to physical or cognitive impairments, the presence of food-related comorbidities such as allergies, intolerances, or medical conditions requiring a specialized diet or artificial nutrition, and presence of neurological or medical conditions unrelated to ASD that could influence food consumption or require specific medical management.

Forty students with ASD attended the school and fourteen (thirteen boys and one girl) met the inclusion criteria and none of the exclusion criteria. All fourteen students agreed to participate in the study and were enrolled. Three participants did not complete the study: two because of repeated school absences with missed sessions that could not be made up within the study period and one because of a transfer to another school. Eleven students completed the study and were included in the final descriptive analyses (Figure 1).

**Figure 1:**
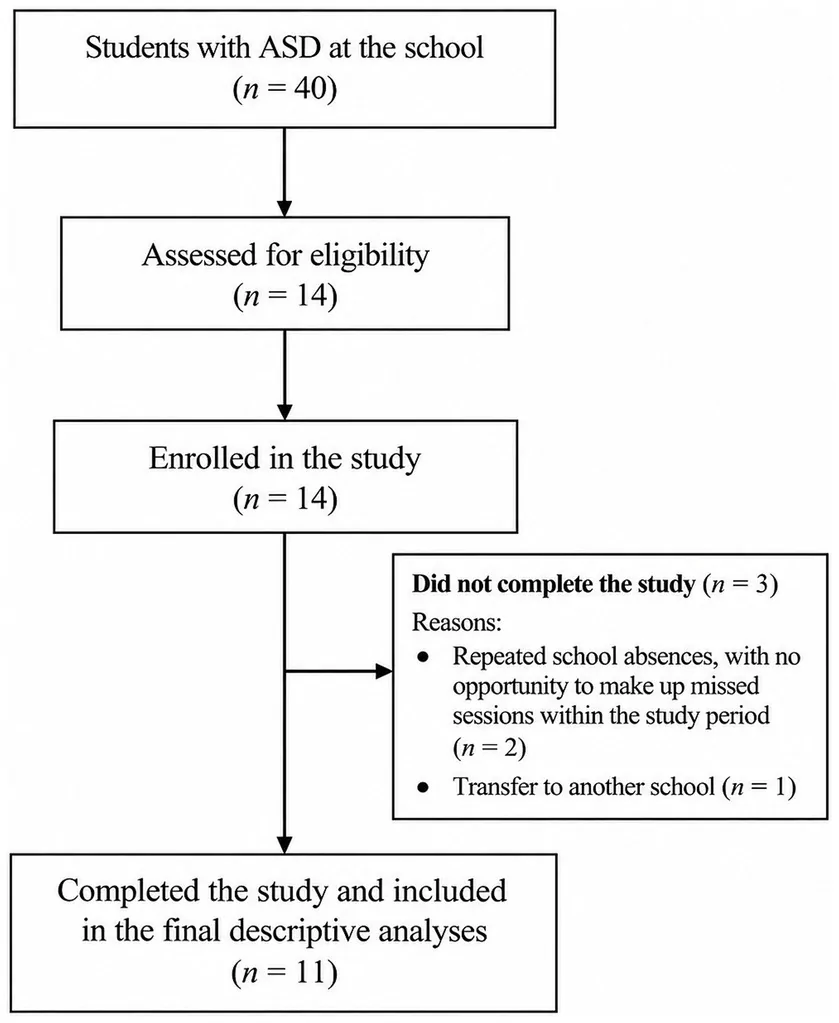
Participant flowchart.

Participation in the project was entirely voluntary, and written informed consent and privacy authorization were provided by parents or legal guardians of the participants before the experimental phase and data collection.

### 2.3 Intervention phase

#### 2.3.1 Sociodemographic information

At baseline, individual sessions with students and their parents were organized to collect participants’ sociodemographic information (data on age, gender, ethnicity, anthropometric measurements, clinical characteristics as the use of medications or dietary supplements). For participants younger than 20 years, BMI-for-age percentiles and weight status categories were determined using the Centers for Disease Control and Prevention (CDC) growth charts. Underweight was defined as BMI-for-age percentiles <5th percentile, normal weight as BMI-for-age percentiles ≥ 5th and <85th percentile, overweight as BMI-for-age percentiles ≥85th and <95th percentile, and obesity as BMI-for-age percentiles ≥ 95th percentile. For the participant aged 20 years, adult BMI cut-offs were applied: underweight (<18.5 kg/m^2^), normal weight (18.5–24.9 kg/m^2^), overweight (25.0–29.9 kg/m^2^), and obesity (≥30.0 kg/m^2^). Each participant was anonymized by assigning a unique number to protect their identity and personal data.

#### 2.3.2 Feeding behaviors during mealtime and adherence to the Mediterranean diet

During the first individual sessions, two questionnaires were administered to evaluate participant feeding-related behaviors using the Screening Tool for Feeding Problems questionnaire (STEP) (26) and the adherence to the MD using the KIDMED questionnaire (27). The STEP questionnaire assesses five areas based on the type of feeding problem addressed by each item: aspiration risk (rumination and vomiting), selectivity (selectivity by food texture, food type, food temperature, setting, and feeder), feeding skills (ability to chew, swallow, or feed independently, and the need for adaptive equipment), food refusal related behavior problems (meal refusal or termination, such as self-injury, aggression, or spitting out food), and nutrition related behavior problems (food stealing, pica, and overeating or undereating) (26). In the present study, the STEP gravity score was not included in the analysis. The KIDMED questionnaire evaluates the adherence to the Mediterranean dietary patterns through 16 items related to different aspects of MD (daily consumption of fruits, vegetables, virgin olive oil, pasta/rice, milk and its derivatives; weekly consumption of fish, legumes, nuts, cereals, and whole food; low consumption of red meat, commercial and processed foods, eggs, butter, sausages, fried and sweet food; tendency to skip breakfast and eat fast food) (27).

After the intervention, the two questionnaires were administered to the same parent for each participant, during individual meetings with the families of the students with ASD.

#### 2.3.3 Lunch sessions and meal service setting

Lunch sessions in the school canteen were planned in collaboration with teachers, support and kitchen staff. The pilot study was structured into two phases in collaboration with the school food service staff. The first phase (T0) evaluated the acceptance of newly introduced meals by students with ASD in the school canteen. The second intervention phase (T1) was designed as a multicomponent intervention consisting of: (a) menu adaptations, including changes to sensory characteristics and food substitutions, to explore the acceptability of the redesigned menus according to sensory characteristics identified in the literature as potentially relevant to individuals with ASD (2, 6, 28); (b) changes in mealtime organisation, including greater autonomy in seating choice and increased opportunities for peer interaction. Parents and support staff were informed in advance of the specific dishes that would be served to the participants during each meal session in T0 and T1. No additional strategies, such as showing participants the menu or photographs of the dishes, preparing them for changes in advance, or maintaining consistent mealtimes and routines, were introduced.

The Pictorial Dietary Assessment Tool (PDAT) (29) was used to evaluate consumption of each dish across 12 school meals per participant (six meals at T0 and six meals at T1); to strengthen the reliability of the dietary assessment, the menus were served in the school canteen for two non-consecutive days during a six-week period, with the help of support staff. Additional sessions were arranged for absent participants to ensure complete data collection. Two researchers observed the students’ behavior during lunch without direct interaction. They used the reference images provided by the PDAT, corresponding to different proportions of food consumed (0%, 25%, 50%, 75%, 100%), to visually estimate the proportion of each dish consumed and record it on the PDAT assessment form.

Researchers with expertise in sensorial analysis tasted and photographed each dish to record its sensory characteristics. These were assessed using the PDAT qualitative meal evaluation form, including colour, odour intensity, texture, and serving temperature. Visual homogeneity was additionally assessed, considering the uniformity of colour and texture and the presence of visually distinguishable food components. At the end of each survey day, the research team convened to align on the evaluation of meal organoleptic properties, ensuring consistency in data interpretation.

After each meal session, data collected through the PDAT were decoded and entered into a database and the individual mean consumption percentage was calculated. These same meal assessments and data processing procedures were applied during T1 phase. In addition to sensory-based food redesign, T1 included a modification of the mealtime environment, allowing participants greater autonomy in seating choice and more opportunities for peer interaction. During the T0 phase, canteen tables were arranged to seat one/two students together with a support assistant following the recommendations of their families; during the T1 phase participants were allowed to choose their seats freely, including at tables seating more than two people. Students’ seating positions at the tables were systematically recorded. Furthermore, smartphone use and the time spent seated (minutes) during the meal were documented for each student by the research team in an observation diary. Meal sessions started at approximately 12:00 p.m. and lasted 30–45 min.

#### 2.3.4 Menu design

In the present study, sensory adaptation was operationalized as the modification of selected sensory and presentation characteristics of nutritionally planned dishes, including texture, colour uniformity, visual appearance, and presentation, as well as the substitution of some foods to achieve the desired sensory characteristics, while maintaining the overall nutritional objectives of the menus.

In the absence of specific Italian dietary recommendations for individuals with ASD in relation to healthy eating guidelines, all menus for T0 and T1 phases were nutritionally planned for the study participants according to “the Recommended Daily Intake of Energy and Nutrients for Italian Population” (30) and the Italian Dietary Guidelines for general population (31), to ensure their nutritional adequacy. A Physical Activity Level (PAL) of 1.4 was assumed for menu planning purposes as this value reflected the PAL of the sample. Individual energy requirements were estimated for each of the eleven participants, and the mean energy requirement was calculated as approximately 2,380 kcal per person/day. The menu was planned for the group and did not correspond to the individual requirements of each participant. The target energy content for school lunches was set at approximately 833 kcal per person, corresponding to 35% of the mean daily energy requirement (31).

Menus were designed in accordance with the MD to provide approximately 50% of energy from carbohydrates, 20% from protein, 30% from fat (with saturated fatty acids <10% of energy) as well as approximately 13 g of dietary fiber. All menus included a first course, a second course with a side dish (cooked or fresh vegetables), and one fresh fruit portion. A short pasta shape was chosen for all menus for swallowing safety and extra virgin olive oil was used as a seasoning.

#### 2.3.5 Training sessions of kitchen staff

Before the T0 phase began, a preliminary meeting was held with the kitchen staff to provide instructions on portion sizes, ingredient selection, cooking methods, and dish presentation. Before the T1 phase, another meeting was organized to inform the staff about the modifications made to the dishes. Serving sizes for each course and nutritional guidelines for menu preparation were provided to all kitchen staff.

### 2.4 Nutritional evaluation of menus

#### 2.4.1 Macronutrient content

A 100 g portion of each dish served was sampled once per week during the T0 and T1 phases. Portions of the same dish collected on different sampling days were pooled to obtain one composite sample per dish. Each composite sample was homogenized using a food processor (Tecator 1,094 Homogenizer, Pbi International). All the homogenized samples were freeze-dried (LIO-10 FP, 5Pascal), ground using a laboratory grinder (GM 200, Retsch GmbH) and stored in a desiccator until chemical analysis. Moisture, crude protein, lipid fraction, total dietary fiber, and ash contents were determined according to Association of Official Analytical Chemists (32) methods. Total carbohydrate content was estimated by difference. All analyses were performed in triplicate, using certified reference materials.

#### 2.4.2 Energy content

The energy content of each menu was calculated from the analytically determined macronutrient composition using the appropriate energy conversion factors (33).

### 2.5 Data analysis

Categorical variables were reported as frequencies and percentages, while continuous variables were summarized as median and interquartile range (IQR). Changes between baseline and post-intervention were described using descriptive statistics. Exploratory Spearman rank correlation coefficients (*rho*) were calculated as descriptive measures of associations between baseline feeding problems (STEP questionnaire) and adherence to the MD (KIDMED questionnaire); no significance tests or *p*-values were used.

Concerning the analysis of menu acceptance, the unit of analysis was meal observation. Each dish was evaluated across 132 meal observations (11 participants × 12 observations), and percentages refer to the proportion of meal observations within each consumption category. One participant did not complete all scheduled meal observations during the intervention phase. Therefore, percentages were calculated using the number of available meal observations as the denominator. To explore the relationship between sensory characteristics of dishes at T0 and T1 and feeding problems, an individual indicator was calculated as the proportion of dishes completely consumed (100% of the portion served). These proportions were summarized according to food selectivity severity categories using median and IQR. Given the exploratory nature of the study and the small sample size, no formal hypothesis testing was performed.

All statistical analyses were performed by an independent researcher who was not involved in the study intervention, meal observations, or data collection.

All data analyses were performed using STATA 18 (Stata Corp., College Station, TX, USA).

## 3 Results

### 3.1 Sociodemographic characteristics

Table 1 shows the main sociodemographic characteristics of the sample.

**Table 1:** Sociodemographic and anthropometric characteristics of the sample (*n* = 11).

Characteristics	Value
Age in years (median, IQR)	17 (16–18)
Males (*n*, %)	10 (90.9)
Italian nationality (*n*, %)	11 (100.0)
Verbality	
Non-verbal (*n*, %)	3 (27.3)
Minimally verbal (*n*, %)	1 (9.1)
Verbal (*n*, %)	7 (63.6)
Weight (kg, median, IQR)	63.0 (57.0–80.0)
Height (m, median, IQR)	1.70 (1.68–1.80)
BMI percentile (*n* = 10, median, IQR)	80.5 (3–90)
BMI in classes	
Underweight (*n*, %)	3 (27.3)
Normal weight (*n*, %)	4 (36.4)
Overweight (*n*, %)	3 (27.3)
Obesity (*n*, %)	1 (9.1)
Clinical characteristics (*n*, %)	
Use of medications and/or dietary supplements	8 (72.7)
Maternal education level (*n*, %)	
Secondary school	1 (9.1)
High school	5 (45.5)
University degree or higher	5 (45.5)
Father’s university degree (*n*, %)	
Secondary school	1 (9.1)
High school	4 (36.5)
University degree or higher	6 (54.5)

The sample included eleven adolescents (median age 17), predominantly male (*n* = 10, 90.9%). All students were Italian. The median BMI percentile was 76 (IQR 3–90). Three adolescents (27.3%) were underweight, four (36.4%) had a normal BMI, and three (27.3%) were overweight. Only one participant (9.1%) was affected by obesity.

Information on ASD severity levels was not available in the participants’ clinical records. Participants were only characterized according to their verbal ability: three students were nonverbal and eight were verbal, in detail, one was minimally verbal and seven were verbal. Eight participants (72.7%) reported using medications and/or dietary supplements. These clinical characteristics were reported for descriptive purposes and were not included in subsequent analyses.

Concerning parental education, 45.5% of mothers (*n* = 5) and 54.5% of fathers (*n* = 6) held a university degree.

### 3.2 Feeding problems and adherence to the Mediterranean diet at baseline

The distribution of feeding problems assessed by the STEP questionnaire and adherence to the MD assessed using the KIDMED questionnaire at baseline are shown in Figure 2. Five participants had absent or minimal feeding problems (45.5%), five students (45.5%) had moderate feeding problems, and one (9.1%) had severe feeding problems, with a median STEP score of 8 (IQR 4–10). Table 2 shows the distribution of feeding problems across the five categories according to the STEP questionnaire subscales. *Food selectivity* and *nutrition-related behavior* subscales appeared to be the most prevalent feeding problems. For *food selectivity* subscale, six participants (54.5%) fell into the severe category and one participant (9.1%) into the moderate category. Consistent with these findings, more than half of the participants (54.5%) were reported to have eaten only selected types of food more than 10 times in the previous month, whereas preferences for a specific eating setting, food temperature, feeder, or food texture were less frequent (Supplementary Table S1). For *nutrition-related behavior problems* subscale (Table 2), five participants (45.5%) were classified in the severe category, one participant (9.1%) in the moderate category and five participants (45.5%) in the absent or minimal category. The most frequent nutrition-related behaviors were continuing to eat as long as food was available, reported for four participants (36.4%) at least once during the previous month, food stealing outside mealtimes, reported for five participants (45.5%) and pica reported for two participants (18.2%) (Supplementary Table S1). *Aspiration risk*, *feeding skills* and *food refusal* problems were not prevalent feeding problems at baseline, as most participants fell into the absent or minimal category (Table 2). However, seven participants (63.6%) reported eating a large amount of food in a short period of time and eight (72.7%) reported swallowing without chewing sufficiently more than 10 times in the previous month. Food refusal behaviors were rare, with only one participant (9.1%) attempting to leave the eating area when food was presented (Supplementary Table S1).

**Figure 2:**
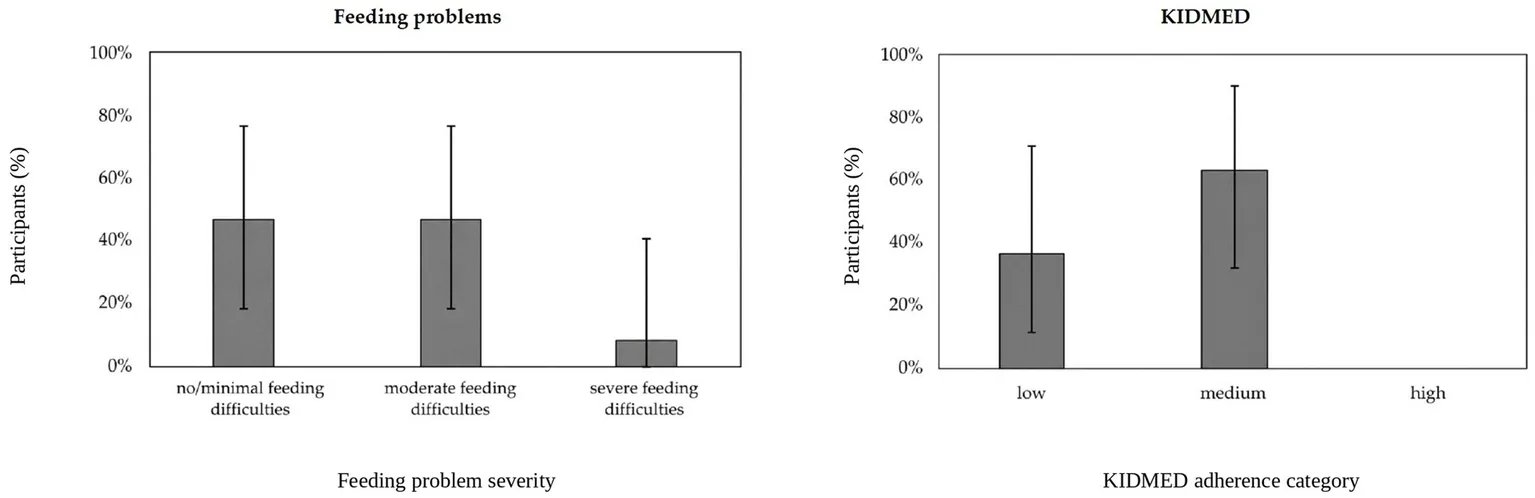
Distribution of feeding problems (STEP questionnaire) and Mediterranean Diet Adherence (KIDMED questionnaire) by level at baseline.

**Table 2:** Distribution of STEP questionnaire subscales among participants at baseline.

STEP questionnaire subscales	*n* (%)	CI 95%
Aspiration risk		
Absent or minimal	11 (100.0)	–
Moderate	0 (0.0)	–
Severe	0 (0.0)	–
Food selectivity		
Absent or minimal	4 (36.4)	10.9–69.2
Moderate	1 (9.1)	2.3–41.3
Severe	6 (54.5)	23.4–83.3
Feeding skills		
Absent or minimal	10 (90.9)	58.7–99.8
Moderate	1 (9.1)	2.3–41.3
Severe	0 (0.0)	–
Food refusal		
Absent or minimal	10 (90.9)	58.7–99.8
Moderate	1 (9.1)	2.3–41.3
Severe	0 (0.0)	–
Nutrition		
Absent or minimal	5 (45.5)	16.7–76.6
Moderate	1 (9.1)	2.3–41.3
Severe	5 (45.5)	16.7–76.6

KIDMED scores reported in Figure 2 showed low adherence to MD in four participants (36.4%), medium adherence in seven participants (63.6%), and no participants in the high adherence category at baseline, with a median score of 4 (IQR 2–5).

Food group consumption and food habits by KIDMED are reported in Supplementary Table S2. Daily consumption of fruit and vegetables was reported, respectively, by seven (63.6%) and by six participants (54.5%), while a second daily serving was consumed by four participants (36.4%) for fruit and by five participants (45.5%) for vegetables. Regular consumption of fish (at least 2–3 times/week) and pulses (more than once/week) was reported by only one participant each (9.1%). Nine participants (81.8%) reported frequent consumption of pasta or rice. Regarding breakfast habits, six participants (54.5%) reported consumption of commercially baked goods or pastries and two participants (18.2%) skipped breakfast.

Exploratory Spearman coefficients indicated positive correlations between KIDMED scores and the STEP feeding skills subscale (*rho* = 0.693), nutrition subscale (*rho* = 0.546), and total STEP score (*rho* = 0.558). These coefficients are reported descriptively and should be interpreted cautiously given the small sample size, as individual observations may substantially influence the estimated correlations.

### 3.3 Menu design and implementation

All menus for the T0 and T1 phases (T0-1, T0-2, T1-1, and T1-2) were designed by researchers according to the established nutritional target and the canteen style. They included a first course, a second course, a side dish, and one portion of fruit. Menus were prepared by the kitchen staff according to the guidelines provided by the researchers (Supplementary Figures S1, S2).

Supplementary Figure S1 shows the T0 menus with all courses. For this phase, first courses included two types of pasta: “whole grain short pasta (*fusilli*) with diced zucchini” and “refined grain short pasta (*penne*) with tomato and basil sauce.”

“Diced zucchini” was chosen to assess the acceptability of a visible vegetable ingredient, and tomato-based sauce was used to evaluate the acceptance of a more homogeneous and creamy vegetable preparation with a predominantly red color.

Regarding second courses, one meat-based and one fish-based dish - “roasted turkey” and “cod fillets with lemon” - were included to assess the acceptability of different protein sources, particularly fish considering the low consumption reported in the KIDMED results (Supplementary Table S2). These dishes were characterized by low-intensity colors - white and beige tones for the fish dish and beige tones for the meat dish. Two mixed-color side dishes were included: “lettuce and cherry tomato salad” and “cooked green beans and carrots.” Two types of fresh fruit with different colors were selected: “diced strawberries” (red) and “sliced kiwi” (green).

In the T1 phase (Supplementary Figure S2), changes to the menus included both selected sensory modifications of the same food and substitutions with different foods to increase visual homogeneity and chromatic uniformity and to provide softer textures compared with T0.

Sensory modifications that retained the same main ingredient included: “diced zucchini” in the T0-1 menu served as “zucchini purée” in the T1-1 menu; “cod fillets with lemon” in the T0-1 menu reformulated into soft “cod fishballs with lemon” in T1-1 menu; “roasted turkey” in the T0-2 menu reformulated as soft “turkey meatballs” in the T1-2 menu. Other changes involved food substitutions: “refined grain short pasta (*penne*)” in the T0-2 menu replaced with potato *gnocchi*, a soft traditional Italian preparation made with potatoes and refined wheat flour, in the T1-2 menu; “lettuce and cherry tomato salad” replaced with a soft and monocoloured “lamb’s lettuce”; “cooked green beans and carrots” replaced with monocoloured “cooked round carrots”; “diced strawberries” in the T0-1 menu replaced with softer “diced kiwi” without the *columella* (the central white and crunchy core) in the T1-1 menu; “sliced kiwi” in the T0-2 menu replaced with very soft, gelatinous “persimmon pulp” in the T1-2 menu.

Kiwi was served peeled; strawberries were served diced, and persimmon was served as skinless pulp.

In summary, whole-grain pasta was included in two first courses on the T0-1 and T1-1 menus; cod was served in two main courses on the T0-1 and T1-1 menus; and white meat (turkey) was used to prepare two main courses on the T0-2 and T1-2 menus. Extra-virgin olive oil was used to season all dishes.

### 3.4 Distribution of participants across consumption categories for each course at T0 and at T1

Ten participants attended all six meal sessions in each phase, while one participant did not complete the last scheduled meal observations during the T1 phase. Changes in the distribution of participants across consumption categories (0%, 25%, 50%, 75%, 100% of the served portion) between T0 and T1 based on their consumption of each dish across repeated servings, are presented in Figure 3. As regards T0-1 menu (Figure 3A), a descriptive shift towards higher consumption categories was observed for the first course after menu modification (T1-1 menu). The proportion of participants classified in the 100% consumption category increased from 50.0% at T0 to 61.5% at T1, while the proportion in the 0% category slightly decreased from 33.3 to 29.2%. For the second course, the proportion in the 100% category increased from 60.6% at T0 to 67.7% at T1, while the proportion in the 0% category decreased from 27.3 to 18.5%. Consumption patterns for the side dish remained relatively stable across the two time points. In contrast, fruit showed a less favorable distribution at T1: the proportion in the 100% category decreased from 40.9 to 35.4%, while that in the 0% category increased from 43.9 to 61.5%. Concerning the menu 2 (Figure 3B), changes in the distribution of consumption categories were observed for the first course, second course, side dish and fruit. As regards the first course, the proportion in the 100% category remained high but slightly decreased from 66.7% at T0 to 63.6% at T1, while that in the 0% category increased from 15.2 to 25.8%. For the second course, the proportion in the 100% category increased from 54.5 to 63.6%, despite a slight increase in the proportion in the 0% category from 21.2 to 28.8%. On the contrary, the proportion in the 100% category for side dish decreased from 54.5 to 40.9%, while that in the 0% category increased from 36.4 to 50.0%. A less favorable pattern was observed for fruit: the proportion in the 100% category decreased from 40.9 to 16.7%, while that in the 0% category increased from 54.5 to 81.8%.

**Figure 3:**
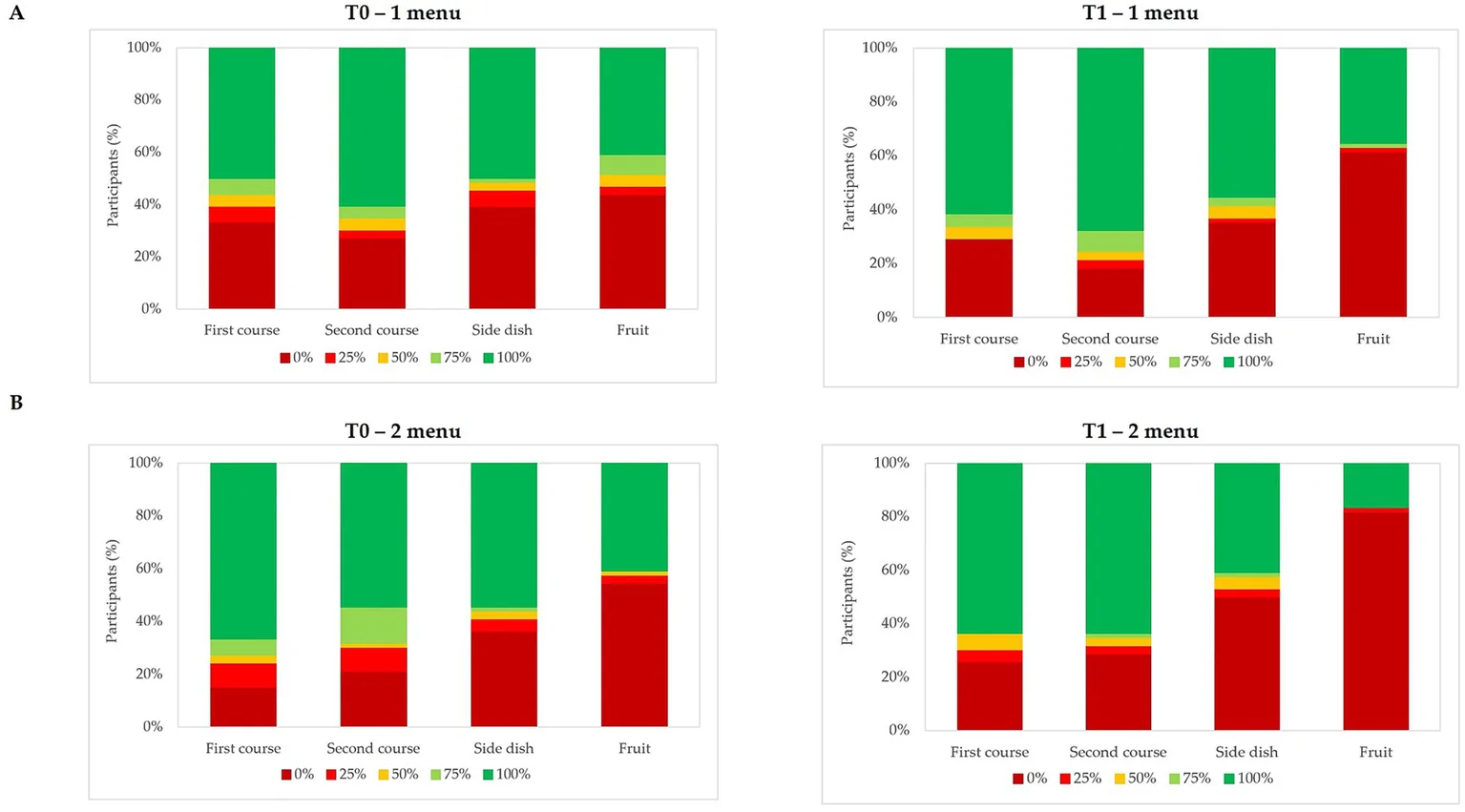
Distribution of meal observations across consumption categories for the study menus. Percentages were calculated based on 132 meal observations per dish at T0 and 131 meal observations per dish at T1. Denominators refer to repeated meal observations rather than individual participants. The difference between TO and T1 was due to incomplete observations for one participant. **(A)** Consumption distribution for Menu 1 at T0 and T1. **(B)** Consumption distribution for Menu 2 at T0 and T1.

### 3.5 Distribution of dish consumption according to sensory characteristics of courses at T0 and T1

Changes in the distribution of participants across consumption categories (0%, 25%, 50%, 75%, and 100% of the served portion), according to dish sensory characteristics at T0 and T1, are shown in Figure 4.

**Figure 4:**
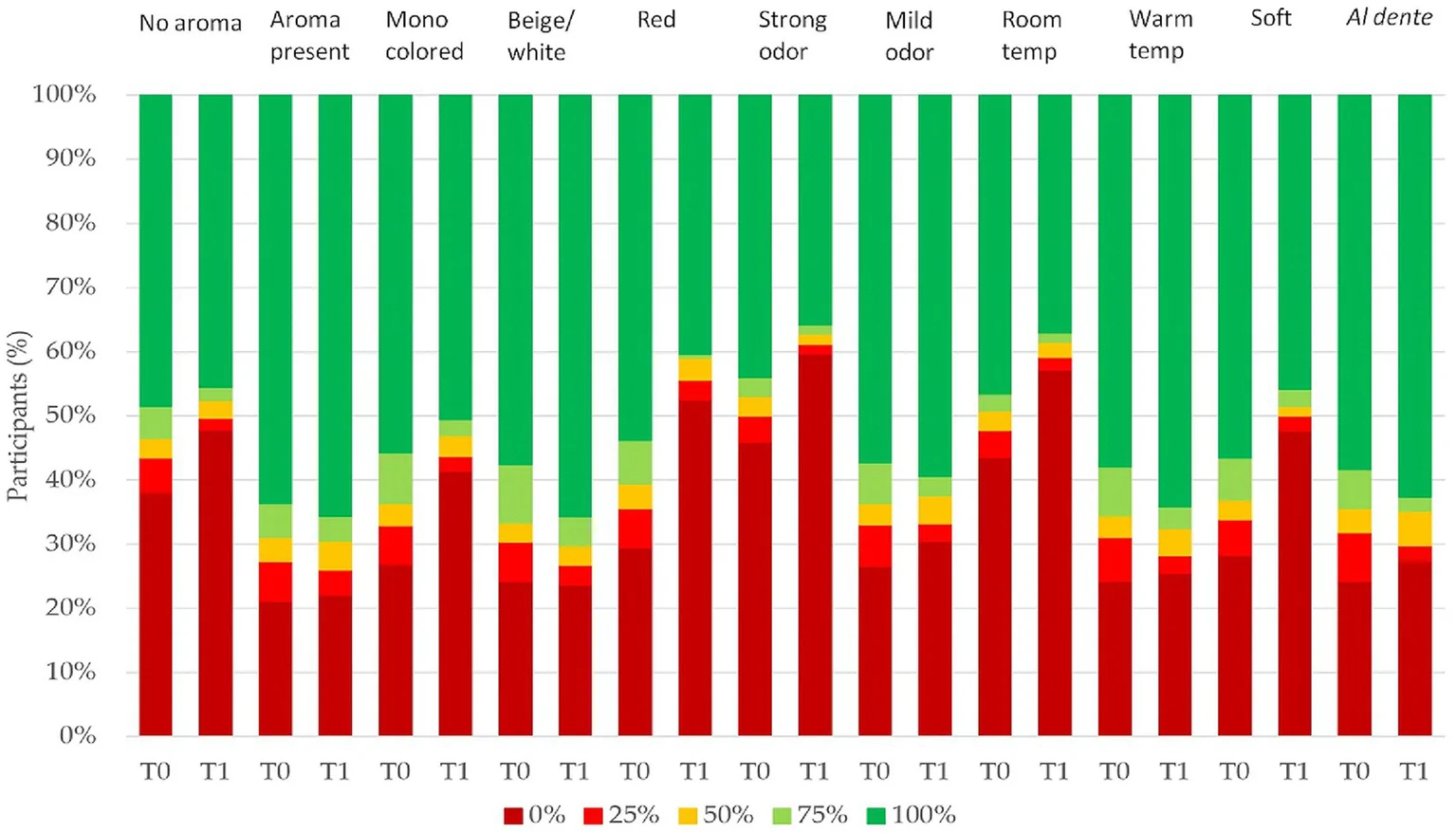
Distribution of meal observations across consumption categories according to the sensory characteristics of the dishes at *T0* and at *T1*. Denominators: No aroma (T0 *n* = 264; T1 *n* = 262), Aroma (T0 *n* = 132; Т1 *n* = 131), Monocolour (T0 *n* = 264; T1 *n* = 524), Beige (T0 *n* = 132; T1 *n* = 131) Red (T0 *n* = 132; T1 *n* = 66) Strong odor (T0 *n* = 198; T1 *n* = 196), Mild odor (T0 *n* = 330; T1 *n* = 328), Room temperature (T0 *n* = 264; T1 *n* = 262) Warm temperature (T0 *n* = 264; T1 *n* =262), Soft texture (T0 *n* = 198; T1 *n* = 262) and al dente texture (T0 *n* = 132; T1 *n* = 65). Sensory categories were not mutually exclusive, and percentages should not be summed.

For each time point, data from menu 1 and menu 2 were pooled for the analysis. At both time points, dishes characterized by the presence of aroma, mild odor, warm temperature, and more homogeneous visual appearance showed higher levels of consumption than dishes with opposite sensory features. At T0, the proportion of participants in the 100% category ranged from 57.3 to 63.6% compared to 43.9 to 48.5% for dishes with opposite characteristics. Similar patterns were observed at T1, with proportions ranging from 59.5 to 65.7% versus 35.7 to 45.6%, respectively.

Between T0 and T1, the proportion of participants in the 100% category increased for warm dishes (from 58.0 to 64.1%) and for dishes with an *al dente* texture (from 58.3 to 62.6%). Conversely, a lower proportion in the 100% category was observed at T1 for dishes with a strong odour (43.9% vs. 35.7%) and red-coloured dishes (53.8% vs. 40.4%), while the proportion in the 0% category increased.

The distribution of consumption categories was broadly similar between T0 and T1 for dishes characterized by the presence of aroma or beige/white coloration. Table 3 reports the median proportion of the fully consumed dishes (100% of the served portion) by each participant according to food selectivity level and the sensory characteristics of the dishes. At T0, participants with absent or minimal food selectivity showed the highest proportions of fully consumed dishes across all sensory characteristics, with median values generally exceeding 90%. In contrast, participants with severe food selectivity showed lower proportions of fully consumed dishes, particularly for dishes without aroma (median 8.3%, IQR 0–75.0), with a strong odor (2.8%, IQR 0–77.8), served at room temperature (6.3%, IQR 0–79.2), with a white/beige color (16.7%, IQR 0–83.3), or with a soft texture (16.7%, IQR 0–83.3). At T1, participants with absent or minimal food selectivity continued to show the highest proportions of fully consumed dishes, with median values ranging from 63.9 to 100%. Among participants with moderate food selectivity, higher median proportions of fully consumed dishes were observed for warm dishes (56.3%), dishes with an *al dente* texture (50.0%), mild-odor dishes (43.4%), and red-colored dishes (45.9%), whereas lower proportions were observed for dishes without aroma (36.1%), served at room temperature (26.9%), and particularly for dishes with a strong odor (11.2%).

**Table 3:** Proportion of fully consumed servings (100%) according to food selectivity score according to dish sensory characteristics.

Sensory characteristcs	STEP: Selectivity subscale					
	Baseline			Post-intervention		
	Absent or minimal (*n* = 4)	Moderate (*n* = 1)	Severe (n = 6)	Absent or minimal (*n* = 2)	Moderate (*n* = 8)	Severe (*n* = 1)
	Median (IQR)	Median (IQR)	Median (IQR)	Median (IQR)	Median (IQR)	Median (IQR)
No aroma	95.85 (89.6–100)	8.3 (8.3–8.3)	8.3 (0–75)	86.1 (83.3–88.9)	36.1 (2.8–77.75)	100 (100–100)
Aroma	95.85 (91.7–100)	25 (25–25)	37.5 (16.7–100)	70.85 (66.7–75)	22.9 (0–58.3)	88.9 (88.9–88.9)
Monocolor	91.7 (83.35–93.75)	20.8 (20.8–20.8)	25 (8.3–83.3)	82.3 (79.2–85.4)	36.5 (2.1–72.9)	97.7 (97.7–97.7)
Beige	95.85 (91.7–100)	41.7 (41.7–41.7)	16.65 (0–83.3)	85 (80–90)	33.35 (1.65–75)	96.4 (96.4–96.4)
Red	91.7 (70.85–91.7)	0 (0–0)	33.35 (16.7–83.3)	83.3 (83.3–83.3)	45.85 (4.15–66.7)	100 (100–100)
Strong odor	86.1 (61.1–88.9)	0 (0–0)	2.8 (0–77.8)	63.85 (44.4–83.3)	11.15 (0–41.65)	100 (100–100)
Mild odor	95 (90–100)	16.7 (16.7–16.7)	21.65 (6.7–86.7)	93.35 (86.7–100)	43.35 (1.65–81.65)	96.4 (96.4–96.4)
Room temperature	87.5 (70.85–89.6)	0 (0–0)	6.25 (0–79.2)	68.75 (58.3–79.2)	26.85 (0–50)	100 (100–100)
Warm temperature	95.85 (89.6–100)	20.8 (20.8–20.8)	22.9 (8.3–87.5)	95.85 (91.7–100)	56.25 (4.15–100)	95.5 (95.5–95.5)
Soft	97.2 (91.65–100)	27.8 (27.8–27.8)	16.65 (0–83.3)	76.4 (72.2–80.6)	31.95 (2.8–63.9)	100 (100–100)
*Al dente*	95.85 (87.5–100)	0 (0–0)	29.2 (16.7–91.7)	100 (100–100)	50 (0–100)	80 (80–80)

### 3.6 Chemical composition and energy content of dishes and menus and macronutrient contributions to total energy at T0 and T1

To assess the nutritional composition of the menus as prepared by the school kitchen staff, macronutrient and energy contents were evaluated (Table 4). Macronutrient contributions to the total energy content of menus at T0 and at T1 per serving were also calculated (Supplementary Figure S3). Carbohydrates contributed 43.1–52.5% of total energy across the four menus, with T0-2 and T1-2 being closest to the planned target of about 50%. For protein, only the T0-2 and T1-1 menus were close to the planned level (22.6 and 22.1% of total energy, respectively), whereas the other two menus exceeded the 20% target. None of the menus reached the planned fat level (30% of total energy), with values ranging from 18.8 to 27.4%. The energy content of the menus ranged from 556 kcal (T0-1) to 672 kcal (T1-2), corresponding to the 66.7–80.7% of recommended total energy content for the menus (833 kcal). Chemical analysis revealed incomplete fidelity between planned nutritional targets and meals produced under routine canteen conditions. No consistent pattern emerged between the fat content of dishes and the distribution of consumption categories. For example, the T1-1 first course showed a reduction in fat content from 8.5% at T0 to 5.1% at T1, while the proportion of fully consumed servings increased from 50.0 to 61.5%. Conversely, the T0-1 side dish showed both an increase in fat content at T1 (from 2.7 to 9.5%) and only a modest increase in full consumption (from 50.0% at T0 to 55.4% at T1). Similarly, in the T0-2/T1-2 comparison, the side dish maintained the same fat content (5.8%) across time points, but the proportion of fully consumed servings decreased from 54.5 to 40.9% (Table 4).

**Table 4:** Chemical composition and energy content of dishes and menus at T0 and T1.

Menu	Dish	Protein (g/serving) mean ± SD	Total fat (g/serving) mean ± SD	Total carbohydrate (g/serving) mean	Total fiber (g/serving) mean ± SD	Ash (g/serving) mean ± SD	Energy content (kcal)
T0-1	Whole grain short pasta (*fusilli*) with diced zucchini	13.1 ± 0.2	8.5 ± 0.2	51.1	12.4 ± 0.2	2.5 ± 0.0	345
	Cod fillets with lemon	25.1 ± 0.6	2.3 ± 0.1	–	–	1.6 ± 0.0	121
	Lettuce and cherry tomato salad	0.7 ± 0.1	2.7 ± 0.1	5.1	1.5 ± 0.5	0.3 ± 0.0	49
	Diced strawberries	1.1 ± 0.2	0.3 ± 0.0	7.7	2.3 ± 0.8	0.5 ± 0.0	41
	TOTAL	40.0	13.8	63.9	16.2	4.9	558
T0-2	Refined grain short pasta (*penne*) with tomato and basil sauce	12.5 ± 0.9	4.2 ± 0.0	62.5	6.4 ± 0.7	2.0 ± 0.2	335
	Roasted turkey	17.3 ± 0.5	2.0 ± 0.1	–	–	1.9 ± 0.0	87
	Cooked carrots and green beans	3.2 ± 0.0	5.8 ± 0.0	9.5	8.6 ± 0.2	3.2 ± 0.9	118
	Sliced kiwi	1.7 ± 0.5	0.8 ± 0.3	13.8	3.6 ± 0.8	0.6 ± 0.2	73
	TOTAL	34.7	12.8	85.8	18.6	7.7	613
T1-1	Whole grain short pasta (*fusilli*) with zucchini purée	11.2 ± 0.2	5.1 ± 0.0	48.6	7.3 ± 0.7	2.4 ± 0.0	288
	Cod fish balls with lemon	21.5 ± 0.1	4.9 ± 0.5	17.3	1.4 ± 0.4	2.2 ± 0.0	198
	Lamb’s lettuce salad	2.1 ± 0.1	9.5 ± 0.0	1.0	2.0 ± 0.1	1.7 ± 0.0	102
	Diced kiwi	1.5 ± 0.5	0.5 ± 0.2	13.4	4.1 ± 0.2	1.1 ± 0.2	69
	TOTAL	36.3	20.0	80.3	14.8	7.4	656
T1-2	Potato *gnocchi* with tomato and basil sauce	5.8 ± 0.6	4.4 ± 0.2	51.8	4.6 ± 0.4	3.0 ± 0.0	266
	Turkey meatballs	35.6 ± 1.0	3.5 ± 0.5	8.1	0.9 ± 0.1	2.5 ± 0.0	206
	Cooked round carrots	1.8 ± 0.0	5.8 ± 0.0	8.6	6.5 ± 0.0	1.4 ± 0.0	105
	Persimmon pulp	0.7 ± 0.5	0.3 ± 0.0	22.1	3.1 ± 0.5	0.9 ± 0.2	95
	TOTAL	43.9	14.0	90.6	15.1	7.8	672

### 3.7 Feeding problems and adherence to Mediterranean diet at post-intervention

Feeding problems assessed by the STEP questionnaire and adherence to the MD assessed by the KIDMED questionnaire at post-intervention are shown in Table 5. Seven participants (63.6%) showed absent or minimal feeding problems, while four (36.4%) had moderate feeding problems. No participants exhibited severe feeding problems. Compared with baseline, a redistribution across STEP feeding problems categories was observed at post-intervention, with the median score changing from 8 (IQR 4–10) to 5 (IQR 4–9). Interestingly, the single participant with severe feeding difficulties at baseline shifted to the moderate category at post-intervention. Table 6 reports STEP subscales scores at post-intervention.

**Table 5:** Feeding problems (STEP questionnaire) and Mediterranean Diet Adherence (KIDMED questionnaire) at post-intervention.

Questionnaire	*n* (%)	CI 95%	Change vs. baseline
Feeding difficulties			
Absent or minimal	7 (63.6)	30.8–89.1	↑
Moderate	4 (36.4)	10.9–69.2	–
Severe	0 (0.0)	–	–
Mediterranean diet adherence			
Low	5 (45.5)	16.7–76.6	↑
Medium	3 (27.3)	6.0–61.0	–
High	3 (27.3)	6.0–61.0	↑

**Table 6:** Feeding problems (STEP questionnaire) subscales of participants at post-intervention.

STEP questionnaire subscales	*n* (%)	CI 95%	Change vs baseline
Aspiration risk			
Absent or minimal	11 (100.0)	–	=
Moderate	0 (0.0)	–	=
Severe	0 (0.0)	–	=
Food selectivity			
Absent or minimal	2 (18.2)	2.3–51.8	↓↓
Moderate	8 (72.7)	39.0–94.0	↑↑
Severe	1 (9.1)	2.3–41.3	↓↓
Feeding skills			
Absent or minimal	11 (100.0)	–	↑
Moderate	0 (0.0)	–	↓
Severe	0 (0.0)	–	=
Food refusal			
Absent or minimal	7 (63.6)	30.8–89.1	↓
Moderate	2 (18.2)	2.3–51.8	↑
Severe	2 (18.2)	2.3–51.8	↑
Nutrition			
Absent or minimal	3 (27.3)	6.0–61.0	↓
Moderate	2 (18.2)	2.3–51.8	↑
Severe	6 (54.5)	23.4–83.3	↑

All participants were classified in the absent or minimal category for *aspiration risk* problems. The greatest redistribution was observed in *food selectivity* subscale. Participants with severe *food selectivity* problems decreased from six to one (54.5% vs. 9.1%), and those with moderate *food selectivity* problems increased from one to eight (9.1% vs. 72.7%), while those with absent or minimal *food selectivity* problems decreased from four to two (36.4% vs. 18.2%). For *feeding skills* subscale, the single participant in the moderate category at baseline shifted to the absent or minimal feeding difficulties category at post-intervention. Conversely, *food refusal related behavior problems* shifted towards higher severity categories: the number of participants in both the moderate and the severe categories increased from one to two (9.1% vs. 18.2%) and from zero to two (18.2%), respectively. Similarly, participants with severe *nutrition-related behavior* problems increased from five to six (45.5% vs. 54.5%).

Supplementary Table S3 reports the distribution of STEP questionnaire responses at post-intervention.

Within the *food selectivity* subscale, eating only selected types of food remained the most frequently reported behavior at both time points, although the proportion of participants reporting this behavior more than 10 times in the previous month decreased from six participants (54.5%) at baseline to five participants (45.5%) at post-intervention. Feeding-skills-related behaviors were descriptively less frequent at post-intervention, including eating a large amount of food in a short period (63.6% vs. 36.4%) and swallowing without sufficient chewing (72.7% vs. 45.5%).

Regarding adherence to MD (Table 5), KIDMED scores at post-intervention showed low adherence in five participants (45.5%), medium adherence in three participants (27.3%), and high adherence in three participants (27.3%). The median score remained unchanged compared with baseline (4, IQR 2–8 vs. 4, IQR 2–5). Notably, three participants (27.3%) shifted from medium to high adherence to MD and consequently, those with medium adherence decreased from seven (63.6%) to three (27.3%). In contrast, participants with low adherence to MD increased from four (36.4%) to five (45.5%).

Figure 5 shows food group consumption and food habits at baseline and at post-intervention according to KIDMED questionnaire. Of note, the number of participants reporting regular fish consumption (at least 2–3/week) increased from one (9.1%) to five (45.5%). The number of participants reporting consumption of commercially baked goods or pastries for breakfast increased from six (54.5%) to ten (90.9%). No changes were observed in the other KIDMED items.

**Figure 5:**
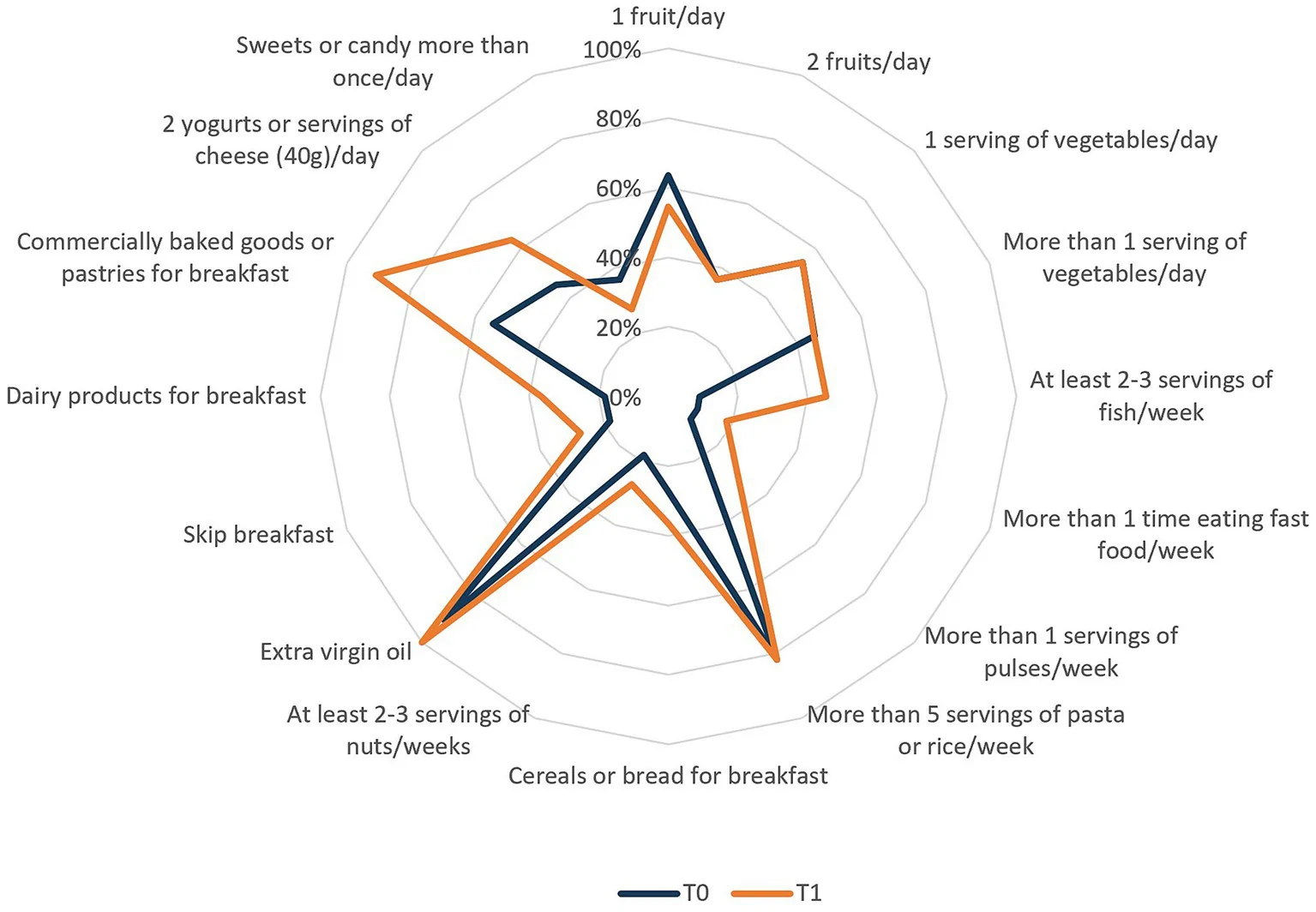
Consumption of food groups and food habits of participants by KIDMED at baseline and at post-intervention.

### 3.8 Mealtime-related behaviors at T0 and at T1

Observations conducted in the school canteen during each mealtime session indicated descriptive changes in behavior from T0 to T1. Supplementary Figure S4 shows the seating distribution of students with ASD during the first mealtime session at T0 and the last mealtime session at T1. During T0 mealtime sessions, students followed a structured table arrangement (two seats per table), and they were accompanied by at least one assistant per student. At T1, tables were arranged to accommodate three or four participants, and they were free to choose their seating: most chose to sit together at shared tables rather than alone. Less frequent excessive smartphone use was observed at T1, with smartphones gradually replaced by food-themed comic books provided by the assistants. During T0, many students tended to leave the table during the meal sessions, whereas during the T1 phase they remained seated at the table with their peers for the entire duration of the meal. More frequent interaction among participants was also observed, with those experiencing fewer behavioral difficulties encouraging and supporting their peers during the meal.

## 4 Discussion

The prevalence of ASD has increased worldwide: an estimated 61·8 million individuals (one in every 127 people) were living with ASD globally in 2021 (34) and a recent meta-analysis reported a prevalence of 0.77% among children 3–18 years (35). The increased prevalence of ASD is probably attributable to greater awareness of the condition, improvements in diagnostic criteria, and enhanced screening and surveillance systems (25, 35–37). Many individuals with ASD develop comorbidities during their lifetime such as obesity and cardiovascular diseases (38) as well as micronutrient deficiencies which may be associated with unhealthy dietary patterns and lifestyles.

Unbalanced diets are common among individuals with ASD and may be partly due to their marked FS (15, 39). Feeding difficulties may be related to sensory processing profiles. Individuals with ASD have deficits in social interaction and social communication as well as restricted, repetitive patterns of behavior, interests, or activity (25) that may contribute to the development of feeding difficulties. Behavioral rigidity and insistence on sameness may lead to a restricted food repertoire, and sensory hyperreactivity may contribute to food aversions (40). Communication difficulties can affect the ability to express hunger, fullness, likes, and dislikes (41). Furthermore, difficulties in social interaction may limit opportunities to learn eating behaviors through observation and participation in shared mealtimes (40). Clinical factors, such as gastrointestinal symptoms and oral-motor difficulties, may also contribute to feeding difficulties and further limit dietary variety (15). Restricted dietary variety may increase the risk of inadequate nutrient intake (1, 42).

These factors can adversely affect health status and impose an additional psychosocial and economic burden on caregivers. FS remains one of the most challenging nutritional issues in ASD because it often persists throughout adolescence and adulthood and substantially affects nutritional status, family routines and quality of life (15, 39, 43).

Consequently, early interventions aimed at improving eating behaviors are strongly recommended (44). School settings may provide a suitable environment to introduce nutritionally planned menus with sensory characteristics adapted to the needs of adolescents with ASD, while supporting healthy eating behaviors and facilitating peer interactions during mealtimes (45). However, evidence regarding school-based nutritional interventions for adolescents with ASD remains scarce (21–23).

In this context, this pilot study is among the first to explore the feasibility and acceptability of sensory-adapted menus for adolescents with ASD in a real-world school canteen setting and to describe mealtime experience in this population. The novelty in our pilot study is that all menus were designed to be nutritionally adequate according to the Italian RDI (30), the Italian Dietary Guidelines (31) and to be consistent with the MD pattern, a dietary pattern widely demonstrated as protective against chronic-degenerative diseases and were subsequently evaluated for their composition as served. To our knowledge, few studies have investigated the adherence to the MD and the prevalence of feeding problems in adolescents with ASD. Moreover, available studies have not yet described the distribution of participants across STEP severity categories or characterized participants according to severity categories within each STEP subscale.

Despite the limited number of participants, the low adherence to MD observed in our study aligns with the lower KIDMED score reported in children and adolescents with ASD compared with typically developing peers (46, 47), suggesting that suboptimal adherence to the Mediterranean dietary pattern may be common in this population, partly because of the greater prevalence of food selectivity among individuals with ASD.

In our study, food selectivity and nutrition-related behavior problems emerged as the predominant feeding problems, with approximately half of the participants falling into the severe category of the STEP subscales. These results provide a detailed characterization of the feeding problems of the participants in our study and are consistent with previous studies showing that a restricted food repertoire is common in individuals with ASD (48, 49).

During the intervention phase, some sensory characteristics were modified including texture, color uniformity, visual appearance and presentation, based on characteristics previously reported as potentially more acceptable for individuals with ASD (8, 29, 50). Following these modifications, higher acceptance was observed for several dishes, particularly for the first course which showed higher acceptance from T0-1 to T1-1 menu, and fish. These dishes were characterized by a mild odor, presence of aroma, warm temperature, and a more homogeneous visual appearance.

The relatively high acceptance of fish dishes among participants suggests that exposure within a structured and supportive environment may facilitate the introduction of some foods that were infrequently consumed at baseline, as previously described in literature in both individuals with ASD and neurotypical ones in other collective catering settings (51, 52). These findings are consistent with evidence that sensory properties can play a crucial role in determining food acceptance among individuals with ASD (52) and that sensory modifications integrated into daily routines may increase the acceptance of some foods (6, 21).

In contrast, fruit consumption remained challenging despite efforts to improve texture and presentation, providing a softer and easier-to-eat form. However, the low acceptability of fruit in the T1 phase may also reflect the substitution of fruits that differed in shape, taste, aroma, acidity, colour, texture, and familiarity to participants, alongside the sensory modifications.

Similar difficulties in increasing fruit and vegetable acceptance have been reported by previous school- and clinic-based interventions (6, 18, 21, 53), suggesting that sensory adaptation alone may not be sufficient for foods, such as fresh fruit, characterized by a substantial variability in taste, acidity, aroma and texture among different products. In particular, fresh fruit presents also intrinsic sensory variability that cannot be easily standardized. Therefore, future research may focus on implementing taste-education workshops specifically designed for children and adolescents with ASD, including structured exposure to fruit with different sensory characteristics, as reported in previous school-based sensory snack interventions (21). Alternative strategies could include different preparation methods, such as incorporating fruit into recipes and developing novel fruit-based products with no added sugars or additives, tailored to the sensory preferences of this population. Moreover, sensory modifications should be evaluated using the same fruit whenever possible, to better distinguish the effect of specific characteristics such as texture or presentation from intrinsic differences between fruit types. These initiatives should include repeated exposure within a structured and supportive environment such as school canteens to increase food consumption. Similarly for side dishes, the stable acceptance at T1 should be interpreted considering the modifications in the types or combinations of vegetables served, rather than sensory adaptations of the same dish. Therefore, differences in consumption may reflect individual food preferences and the characteristics of the vegetables offered, rather than the effects of sensory modifications alone.

The analysis of the sensory characteristics of dishes provides further insight into the mechanisms underlying food acceptance. Across both intervention phases, higher consumption percentages were observed for dishes characterized by mild odor, warm serving temperature, and a more homogeneous visual appearance compared to dishes with stronger odors, or greater visual complexity. These observations are consistent with previous literature describing atypical sensory responsiveness as one of the principal determinants of FS in ASD (3, 4). Although causal inferences cannot be drawn because of the exploratory study design, these findings suggest that relatively simple modifications in meal presentation may represent a feasible approach to address sensory barriers during school meals. However, participants with more severe FS showed lower proportions of completely consumed dishes across almost all sensory categories, a pattern consistent with the potential influence of sensory processing differences on eating behavior in individuals with ASD.

Another relevant finding concerns the descriptive changes observed in feeding behaviors at post-intervention. Although the limited sample size precluded formal statistical comparisons, post-intervention STEP scores suggested an overall shift toward lower categories of feeding difficulties, particularly in the *food selectivity* subscale. However, these changes were not consistent across all STEP subscales, as less favorable patterns were observed for *food refusal* and *nutrition-related behaviors* subscales. Considering that FS often persists over time and may become increasingly resistant to change during adolescence (1, 10), even modest changes may be noteworthy. Given the small sample size and uncontrolled design, these findings should be interpreted as exploratory within-participant observations rather than evidence of intervention effectiveness. Unlike most previous interventions conducted in specialized clinical settings, this study suggests that similar approaches may also be feasible in routine school canteen settings through simple modifications. Their effectiveness should be evaluated in larger controlled studies.

At the end of this study, some changes in dietary habits within the home setting were observed, including higher reported weekly fish consumption and a shift from medium to high adherence to the MD in three participants, whereas one participant shifted towards lower adherence to the MD. These findings are consistent with the relatively high acceptance of the fish-based dishes observed during the study and align with previous studies showing that sensory-based food modifications can enhance acceptance and consumption of specific food groups in children with ASD, and support mealtime-related behaviors in school-based settings (21, 22). However, the median KIDMED score remained unchanged, and changes in individual KIDMED items were heterogeneous. Therefore, these observations cannot be interpreted as evidence of an overall improvement in MD adherence. Longer intervention and follow-up periods may be required to achieve measurable changes in dietary patterns within the home setting.

Our exploratory pilot study also focused on assessing the nutritional value of the dishes served to participants in the school canteen. To our knowledge, few studies have analysed the nutritional composition of served menus in school canteens (53). The nutritional evaluation of the served menus showed lower energy content than the target provided to kitchen staff; protein exceeded the planned proportion of energy in two menus, whereas fat was below the planned level in all four menus. These deviations highlight the challenges of translating nutritional plans into meals prepared under routine canteen conditions, despite specific staff training.

Canteen staff may have encountered multiple constraints related to staffing, time and resources that limit full adherence to the planned nutritional targets as described in a recent review (54). More structured monitoring and support strategies, including standardized preparation protocols and periodic supervision by nutritionists, may improve compliance with nutritional guidelines. Such monitoring should accompany the sensory adaptation of menus to support the provision of nutritionally adequate menus that are also acceptable to adolescents with ASD because of their sensory characteristics. Our findings support a more integrated approach to school menu design that combines nutritional planning with sensory adaptations tailored to students with ASD. However, given the multicomponent nature of the intervention, the observed changes in food acceptance and mealtime-related behaviors may reflect the combined influence of sensory-based menu modifications, food substitutions and mealtime organisation. Their individual contributions cannot be determined within the present study design.

Finally, noteworthy observations emerged regarding participants’ social behaviors during mealtimes. Mealtimes can represent a source of stress and social discomfort for individuals with ASD due to feeding difficulties, potentially leading to avoidance behaviors and limited participation in shared meals (10, 55). In our pilot study, increased social interaction, engagement and peer support, as well as fewer disruptive behaviors (e.g., leaving the table and excessive smartphone use) were observed at T1. These results should be interpreted in light of the change in seating arrangements during the T1 phase, when participants were free to choose where and with whom to sit, thereby providing greater opportunities for peer interaction. Overall, these observations suggest that allowing adolescents with ASD to choose where and with whom to sit may facilitate social participation and positive peer interactions during school mealtimes.

### 4.1 Limitations and strengths of the study

Although the methods and findings of our study provide useful insights into the feasibility of implementing sensory-adapted menus for adolescents with ASD in school canteen settings, the study has several limitations. First, the small sample size resulted from difficulties in enrolling eligible students with ASD attending the school. Many students were unable to eat independently due to physical or cognitive impairments, or had food-related comorbidities such as allergies, intolerances, or medical conditions requiring a specialized diet. Two students did not complete the study because they were unable to consistently attend the lunch sessions scheduled at 12:00 p.m. This change to the usual school timetable was necessary to meet the school’s organisational needs, including the preparation of study meals, and enable the study to be conducted. The study in Rome was conducted at a single participating school rather than the two originally registered, which may limit the generalizability of the findings to other school canteen settings.

Another limitation was the absence of a control group which limits the internal validity of the study and precludes causal inferences from being drawn regarding the relationship between the intervention and the observed outcomes. The predominantly male sample limits the generalizability of the findings to female adolescents with ASD and information on participants’ DSM-5 ASD severity levels (Levels 1–3) was unavailable in the clinical records. Consequently, we were unable to examine whether food acceptance or mealtime-related responses differed according to participants’ ASD severity levels.

All participants underwent the T0 phase followed by the T1 phase with repeated meal exposures during each phase. These repeated sessions may have increased participants’ familiarity with the researchers, study procedures, canteen environment, and foods served, potentially influencing food acceptance and mealtime-related behaviors.

Finally, the relatively short duration of the study (4 months) and the limited number of meals served precluded assessment of long-term changes in dietary habits and FS.

Consequently, the findings of this study should be interpreted as exploratory and descriptive, focusing on feasibility and acceptability rather than definitive intervention effectiveness.

Future studies with larger sample sizes, a more balanced sex distribution and controlled designs are needed to evaluate the effectiveness of sensory-adapted menu interventions in school canteens. Longer follow-up periods and repeated exposure to tailored meals would help clarify behavioral and dietary changes and their potential long-term impact on health outcomes.

Despite these limitations, our pilot study has several strengths. First, although the number of participants was limited, the study included repeated direct observations of food consumption across multiple school meals for each participant. This approach provided a detailed description of individual consumption patterns in a real-world canteen setting.

Second, the study was conducted in a real school environment, a key setting for adolescents with ASD. It provides preliminary insights into the feasibility of implementing sensory-adapted menus in schools and documents the mealtime experience among adolescents with ASD in a routine canteen setting. During the second phase of the study, participants were allowed to choose their seating and spontaneous peer interactions were observed more frequently during mealtimes. A participant-centered approach was adopted, involving repeated interactions with families and school staff. This collaborative process has supported the implementation of the intervention.

Another strength of this study is that, to our knowledge, this is one of the few studies to have nutritionally planned school menus and subsequently assessed the composition of the meals as served and their compliance with the recommendations provided to canteen staff.

This finding underscores the importance of adequately training and supporting school catering staff to improve adherence to nutritional targets and the overall quality of the mealtime experience. Meals were prepared and consumed within the routine school setting, enhancing the ecological validity and potential scalability of the intervention. In addition, data collection used a non-invasive, low-burden approach suitable for adolescents with ASD.

Overall, although methodological constraints preclude causal inference, the results of this pilot study support the real-world feasibility of implementing sensory-adapted, nutritionally planned menus in school canteens for adolescents with ASD.

## 5 Conclusion

Overall, despite its limitations, this exploratory pilot study provides preliminary insights into the feasibility of implementing a multicomponent intervention combining sensory-adapted menus, and mealtime interventions for adolescents with ASD in a real-world school canteen. To our knowledge, this is one of the few studies conducted in a school canteen setting involving individuals with ASD that considers not only modifications to the sensory characteristics of the menus but also their nutritional planning and the subsequent assessment of the meals as served. Relatively high consumption was observed for some dishes, particularly first and second courses, whereas fruit acceptance remained challenging. Changes in mealtime-related social behaviors were also documented, including less frequent smartphone use and a greater tendency among participants to sit and eat together with peers. However, these observations coincided with a change in seating arrangements and cannot be attributed specifically to menu adaptation.

Although the results of this study should be interpreted as context-specific and exploratory, three elements appeared to be particularly relevant for future research: (i) the feasibility of introducing sensory-adapted, nutritionally planned menus for adolescents with ASD in routine canteen services; (ii) the potential role of a supportive school setting in facilitating peer interaction; (iii) the importance of standardized preparation protocols, monitoring, and periodic supervision by nutritionists to improve fidelity to nutritional targets for canteen staff.

These elements should be considered in the design of larger controlled intervention studies aimed at better understanding food selectivity and supporting mealtime-related social behaviors and the overall mealtime experience among children and adolescents with ASD in school canteen settings.

## Data Availability

The raw data supporting the conclusions of this article will be made available by the authors, without undue reservation.
