# Supplementary material for: Exploring the feasibility and acceptability of sensory-adapted menus for adolescents with autism spectrum disorder in a school canteen: the AUT-MENU pilot study
Source: Front Nutr. 2026 Oct 30;13:1978351. doi: 10.3389/fnut.2026.1978351 (PMC13759793; doi:10.3389/fnut.2026.1978351)
Supplement: Supplementary file 1 [file Supplementary_file_1.DOCX]

Supplementary Material

**Table S1**. **Distribution of STEP questionnaire responses at baseline.**

|  | **Baseline** | |
| --- | --- | --- |
|  | n (%) | 95% CI |
| **Aspiraton Risk** |  |  |
| Regurgitates and reswallows food either during or immediately following meals |  |  |
| *Never* | 11 (100.0%) | - |
| *1-10 times in the last month* | 0 (0.0%) | - |
| *More than 10 times in the last month* | 0 (0.0%) | - |
| Vomits either during or immediately following meals |  |  |
| *Never* | 11 (100.0%) | - |
| *1-10 times in the last month* | 0 (0.0%) | - |
| *More than 10 times in the last month* | 0 (0.0%) | - |
| **Selectivity** |  |  |
| Will only eat selected types of food |  |  |
| *Never* | 5 (45.5%) | 16.7 – 76.6 |
| *1-10 times in the last month* | 0 (0.0%) | - |
| *More than 10 times in the last month* | 6 (54.5%) | 23.4 – 83.3 |
| Prefers a certain setting for eating |  |  |
| *Never* | 8 (72.7%) | 39.0 – 94.0 |
| *1-10 times in the last month* | 2 (18.2%) | 2.3 – 51.8 |
| *More than 10 times in the last month* | 1 (9.1%) | 2.3 – 41.3 |
| Will only eat foods of a certain temperature |  |  |
| *Never* | 9 (81.8%) | 48.2 – 97.7 |
| *1-10 times in the last month* | 1 (9.1%) | 2.3 – 41.3 |
| *More than 10 times in the last month* | 1 (9.1%) | 2.3 – 41.3 |
| Prefers to be fed by a specific caregiver, or prefers to be fed rather than independently feed |  |  |
| *Never* | 9 (81.8%) | 48.2 – 97.7 |
| *1-10 times in the last month* | 1 (9.1%) | 2.3 – 41.3 |
| *More than 10 times in the last month* | 1 (9.1%) | 2.3 – 41.3 |
| Eats food of only certain texture |  |  |
| *Never* | 8 (72.7%) | 39.0 – 94.0 |
| *1-10 times in the last month* | 2 (18.2%) | 2.3 – 51.8 |
| *More than 10 times in the last month* | 1 (9.1%) | 2.3 – 41.3 |
| **Skills** |  |  |
| Individual cannot feed him/herself independently |  |  |
| *Never* | 10 (90.9%) | 58.7 – 99.8 |
| *1-10 times in the last month* | 1 (9.1%) | 2.3 – 41.3 |
| *More than 10 times in the last month* | 0 (0.0%) | - |
| Does not demonstrate the ability to chew |  |  |
| *Never* | 11 (100.0%) | - |
| *1-10 times in the last month* | 0 (0.0%) | - |
| *More than 10 times in the last month* | 0 (0.0%) | - |
| Chokes on food |  |  |
| *Never* | 10 (90.9%) | 58.7 – 99.8 |
| *1-10 times in the last month* | 1 (9.1%) | 2.3 – 41.3 |
| *More than 10 times in the last month* | 0 (0.0%) | - |
| Does not demonstrate the ability to swallow |  |  |
| *Never* | 11 (100.0%) | - |
| *1-10 times in the last month* | 0 (0.0%) | - |
| *More than 10 times in the last month* | 0 (0.0%) | - |
| Requires special equipment for feeding |  |  |
| *Never* | 11 (100.0%) | - |
| *1-10 times in the last month* | 0 (0.0%) | - |
| *More than 10 times in the last month* | 0 (0.0%) | - |
| Eats a large amount of food in a short period of time |  |  |
| *Never* | 4 (36.4%) | 10.9 – 69.2 |
| *1-10 times in the last month* | 0 (0.0%) | - |
| *More than 10 times in the last month* | 7 (63.6%) | 30.8 – 89.1 |
| Requires special positioning during feeding |  |  |
| *Never* | 11 (100.0%) | - |
| *1-10 times in the last month* | 0 (0.0%) | - |
| *More than 10 times in the last month* | 0 (0.0%) | - |
| Swallows without chewing sufficiently |  |  |
| *Never* | 3 (27.3%) | 6.0 – 61.0 |
| *1-10 times in the last month* | 0 (0.0%) | - |
| *More than 10 times in the last month* | 8 (72.7%) | 39.0 – 94.0 |
| **Refusal** |  |  |
| Problem behaviors increase during mealtime | 11 (100.0%) | - |
| *Never* | 0 (0.0%) | - |
| *1-10 times in the last month* | 0 (0.0%) | - |
| *More than 10 times in the last month* |  |  |
| Spits out their food before swallowing |  |  |
| *Never* | 11 (100.0%) | - |
| *1-10 times in the last month* | 0 (0.0%) | - |
| *More than 10 times in the last month* | 0 (0.0%) | - |
| Pushes food away or attempts to leave the area when food is presented |  |  |
| *Never* | 10 (90.9%) | 58.7 – 99.8 |
| *1-10 times in the last month* | 1 (9.1%) | 2.3 – 41.3 |
| *More than 10 times in the last month* | 0 (0.0%) | - |
| **Nutrition** |  |  |
| Steals or attempts to steal food from others during mealtime |  |  |
| *Never* | 9 (90.9%) | 58.7 – 99.8 |
| *1-10 times in the last month* | 1 (9.1%) | 2.3 – 41.3 |
| *More than 10 times in the last month* | 0 (0.0%) | - |
| Eats or attempts to eat items that are not food |  |  |
| *Never* | 9 (81.8%) | 48.2 – 97.7 |
| *1-10 times in the last month* | 2 (18.2%) | 2.3 – 51.8 |
| *More than 10 times in the last month* | 0 (0.0%) | - |
| Only eats a small amount of the food presented |  |  |
| *Never* | 9 (81.8%) | 48.2 – 97.7 |
| *1-10 times in the last month* | 2 (18.2%) | 2.3 – 51.8 |
| *More than 10 times in the last month* | 0 (0.0%) | - |
| Will continue to eat as long as food is available |  |  |
| *Never* | 7 (63.6%) | 30.8 – 89.1 |
| *1-10 times in the last month* | 2 (18.2%) | 2.3 – 51.8 |
| *More than 10 times in the last month* | 2 (18.2%) | 2.3 – 51.8 |
| Steals or attempts to steal food outside of mealtime |  |  |
| *Never* | 6 (54.5%) | 23.4 – 83.3 |
| *1-10 times in the last month* | 2 (18.2%) | 2.3 – 51.8 |
| *More than 10 times in the last month* | 3 (27.3%) | 6.0 – 61.0 |

**Table S2. Food group consumption and food habits of participants according to KIDMED questionnaire at baseline.**

| **KIDMED questionnaire** | **n (%)** | **CI 95%** |
| --- | --- | --- |
| Fruit or fruit juice daily | 7 (63.6) | 30.8 – 89.1 |
| Second serving of fruit daily | 4 (36.4) | 10.9 – 69.2 |
| Fresh or cooked vegetables daily | 6 (54.5) | 23.4 – 83.3 |
| Fresh or cooked vegetables > 1/day | 5 (45.5) | 16.7 – 76.6 |
| Regular fish consumption (at least 2–3/week) | 1 (9.1) | 2.3 – 41.3 |
| > 1/week fast-food (hamburger) restaurant | 1 (9.1) | 2.3 – 41.3 |
| Pulses > 1/week | 1 (9.1) | 2.3 – 41.3 |
| Pasta or rice almost daily (> 5/week) | 9 (81.8) | 48.2 – 97.7 |
| Cereal or cereal product for breakfast | 3 (27.3) | 6.0 – 61.0 |
| Regular nut consumption (at least 2–3/week) | 2 (18.2) | 2.3 – 51.8 |
| Use of olive oil at home | 10 (90.9) | 58.7 – 99.8 |
| Skip breakfast | 2 (18.2) | 2.3 – 51.8 |
| Dairy product for breakfast | 2 (18.2) | 2.3 – 51.8 |
| Commercially baked goods or pastries for breakfast | 6 (54.5) | 23.4 – 83.3 |
| Two yoghurts and/or 40 g cheese daily | 5 (45.5) | 16.7 – 76.6 |
| Sweets and candy several times a day | 4 (36.4) | 10.9 – 69.2 |

**Figure S1.** **T0 phase menus composition.**

**Figure S2. T1 phase menus composition.**

**Table S3. Distribution of STEP questionnaire responses at post-intervention.**

|  | **Post-intervention** | |
| --- | --- | --- |
|  | n (%) | 95% CI |
| **Aspiraton Risk** |  |  |
| Regurgitates and reswallows food either during or immediately following meals |  |  |
| *Never* | 11 (100.0%) | - |
| *1-10 times in the last month* | 0 (0.0%) | - |
| *More than 10 times in the last month* | 0 (0.0%) | - |
| Vomits either during or immediately following meals |  |  |
| *Never* | 11 (100.0%) | - |
| *1-10 times in the last month* | 0 (0.0%) | - |
| *More than 10 times in the last month* | 0 (0.0%) |  |
| **Selectivity** |  |  |
| Will only eat selected types of food |  |  |
| *Never* | 5 (45.4%) | 16.7 – 76.6 |
| *1-10 times in the last month* | 1 (9.1%) | 2.3 – 41.3 |
| *More than 10 times in the last month* | 5 (45.4%) | 16.7 – 76.6 |
| Prefers a certain setting for eating |  |  |
| *Never* | 10 (90.9%) | 58.7 – 99.8 |
| *1-10 times in the last month* | 0 (0.0%) | - |
| *More than 10 times in the last month* | 1 (9.1%) | 2.3 – 41.3 |
| Will only eat foods of a certain temperature |  |  |
| *Never* | 10 (90.9%) | 58.7 – 99.8 |
| *1-10 times in the last month* | 1 (9.1%) | 2.3 – 41.3 |
| *More than 10 times in the last month* | 0 (0.0%) | - |
| Prefers to be fed by a specific caregiver, or prefers to be fed rather than independently feed |  |  |
| *Never* | 8 (72.7%) | 39.0 – 94.0 |
| *1-10 times in the last month* | 1 (9.1%) | 2.3 – 41.3 |
| *More than 10 times in the last month* | 2 (18.2%) | 2.3 – 51.8 |
| Eats food of only certain texture |  |  |
| *Never* | 10 (90.9%) | 58.7 – 99.8 |
| *1-10 times in the last month* | 1 (9.1%) | 2.3 – 41.3 |
| *More than 10 times in the last month* | 0 (0.0%) | - |
| **Skills** |  |  |
| Individual cannot feed him/herself independently |  |  |
| *Never* | 11 (100.0%) | - |
| *1-10 times in the last month* | 0 (0.0%) | - |
| *More than 10 times in the last month* | 0 (0.0%) | - |
| Does not demonstrate the ability to chew |  |  |
| *Never* | 11 (100.0%) | - |
| *1-10 times in the last month* | 0 (0.0%) | - |
| *More than 10 times in the last month* | 0 (0.0%) | - |
| Chokes on food |  |  |
| *Never* | 11 (100.0%) | - |
| *1-10 times in the last month* | 0 (0.0%) | - |
| *More than 10 times in the last month* | 0 (0.0%) | - |
| Does not demonstrate the ability to swallow |  |  |
| *Never* | 11 (100.0%) | - |
| *1-10 times in the last month* | 0 (0.0%) | - |
| *More than 10 times in the last month* | 0 (0.0%) | - |
| Requires special equipment for feeding |  |  |
| *Never* | 11 (100.0%) | - |
| *1-10 times in the last month* | 0 (0.0%) | - |
| *More than 10 times in the last month* | 0 (0.0%) | - |
| Eats a large amount of food in a short period of time |  |  |
| *Never* | 6 (54.5%) | 23.4 – 83.3 |
| *1-10 times in the last month* | 1 (9.1%) | 2.3 – 41.3 |
| *More than 10 times in the last month* | 4 (36.4%) | 10.9 – 69.2 |
| Requires special positioning during feeding |  |  |
| *Never* | 11 (100.0%) | - |
| *1-10 times in the last month* | 0 (0.0%) | - |
| *More than 10 times in the last month* | 0 (0.0%) | - |
| Swallows without chewing sufficiently |  |  |
| *Never* | 4 (36.4%) | 10.9 – 69.2 |
| *1-10 times in the last month* | 2 (18.2%) | 2.3 – 51.8 |
| *More than 10 times in the last month* | 5 (45.4%) | 16.7 – 76.6 |
| **Refusal** |  |  |
| Problem behaviors increase during mealtime | 9 (81.8%) | 48.2 – 97.7 |
| *Never* | 1 (9.1%) | 2.3 – 41.3 |
| *1-10 times in the last month* | 1 (9.1%) | 2.3 – 41.3 |
| *More than 10 times in the last month* |  |  |
| Spits out their food before swallowing |  |  |
| *Never* | 11 (100.0%) | - |
| *1-10 times in the last month* | 0 (0.0%) | - |
| *More than 10 times in the last month* | 0 (0.0%) | - |
| Pushes food away or attempts to leave the area when food is presented |  |  |
| *Never* | 9 (81.8%) | 48.2 – 97.7 |
| *1-10 times in the last month* | 1 (9.1%) | 2.3 – 41.3 |
| *More than 10 times in the last month* | 1 (9.1%) | 2.3 – 41.3 |
| **Nutrition** |  |  |
| Steals or attempts to steal food from others during mealtime |  |  |
| *Never* | 9 (81.8%) | 48.2 – 97.7 |
| *1-10 times in the last month* | 2 (18.2%) | 2.3 – 51.8 |
| *More than 10 times in the last month* | 0 (0.0%) | - |
| Eats or attempts to eat items that are not food |  |  |
| *Never* | 10 (90.9%) | 58.7 – 99.8 |
| *1-10 times in the last month* | 1 (9.1%) | 2.3 – 41.3 |
| *More than 10 times in the last month* | 0 (0.0%) | - |
| Only eats a small amount of the food presented |  |  |
| *Never* | 10 (90.9%) | 58.7 – 99.8 |
| *1-10 times in the last month* | 1 (9.1%) | 2.3 – 41.3 |
| *More than 10 times in the last month* | 0 (0.0%) | - |
| Will continue to eat as long as food is available |  |  |
| *Never* | 7 (63.6%) | 30.8 – 89.1 |
| *1-10 times in the last month* | 1 (9.1%) | 2.3 – 41.3 |
| *More than 10 times in the last month* | 3 (27.3%) | 6.0 – 61.0 |
| Steals or attempts to steal food outside of mealtime |  |  |
| *Never* | 6 (54.5%) | 23.4 – 83.3 |
| *1-10 times in the last month* | 3 (27.3%) | 6.0 – 61.0 |
| *More than 10 times in the last month* | 2 (18.2%) | 2.3 – 51.8 |

**Figure S3.** Macronutrients contribution (%) to Total Energy Content of T0 and T1 menus.

T0-1

|  | T0-1 Menu |
| --- | --- |
| Protein | 28.8 |
| Lipid | 22.3 |
| Carbohydrates | 43.1 |

T1-1

|  | T1-1 Menu |
| --- | --- |
| Protein | 22.1 |
| Lipid | 27.4 |
| Carbohydrates | 45.9 |

T0-2

|  | T0-2 Menu |
| --- | --- |
| Protein | 22.6 |
| Lipid | 18.8 |
| Carbohydrates | 52.5 |

T1-2

|  | T1-2 Menu |
| --- | --- |
| Protein | 26.1 |
| Lipid | 18.8 |
| Carbohydrates | 50.6 |

**Figure S4.** Participants seating arrangement during meals at T0 and at T1.
