# Supplementary material for: Exploring the feasibility and acceptability of sensory-adapted menus for adolescents with autism spectrum disorder in a school canteen: the AUT-MENU pilot study
Source: Front Nutr. 2026 Oct 30;13:1978351. doi: 10.3389/fnut.2026.1978351 (PMC13759793; doi:10.3389/fnut.2026.1978351)
Supplement: Supplementary file 2 [file Supplementary_file_2.docx]

# CONSERVE Checklists

| CONSERVE-CONSORT Extension: 01/09/2026 | | | | | |
| --- | --- | --- | --- | --- | --- |
| Item | Item Title | Description | | | Manuscript section |
| I. | Extenuating Circumstances | The start of the Rome school-based component of the AUT-MENU study was delayed because the ethical approval process required more time than originally anticipated. Study activities involving participants could not begin before formal approval by the competent Ethics Committee. Consequently, the number of planned meal observations per participant was reduced from 16 to 12 to accommodate participants' attendance at the school canteen.  One of the two registered Rome schools (IPSEOA “Tor Carbone” high school) could not be included because an on-site canteen service, required to implement the planned study activites, was not available.  The upper age limit was extended to 20 years to allow the inclusion of eligible students attending the participating school. | | | Materials and Methods - Study Design and Setting |
| II. | Important Modifications | a. Important modifications at the Rome school-based component of the AUT-MENU study included:  - postponement of the study timeline - conduct of the school component at the Giuseppe Garibaldi Agricultural Technical Institute only - reduction of planned meal assessments from 16 to 12 per participant (six at T0 and six at T1) - extension of the upper age limit to 20 years to allow participation of students with ASD who were still regularly enrolled at the participating school. | | | Materials and Methods - Study Design and Setting |
|  |  | b. Direct impacts included delayed start of the Rome school-based component of the AUT-MENU study and the inability to implement the study at the second registered Rome school because no canteen was available. As a feasibility-oriented response to the shortened school timeframe and participants' attendance schedules, the investigators adapted the meal-assessment schedule from 16 to 12 assessments per participant, equally distributed between T0 and T1. This reduced the number of repeated meal observations but allowed both study phases to be completed within the school setting. The age eligibility for the Rome school component was also adapted to include 20-year-old students with ASD who were regularly enrolled. The primary food-acceptance outcome and the sensory and nutritional menu-adaptation approach were not changed. | | |  |
|  |  | c. The modification occurred before the start of the study activities in Rome. Ethical approval was obtained on 30 October 2025 and study activities started in November 2025 and were completed in February 2026. | | | Materials and Methods - Study Design and Setting |
| III. | Responsible Parties | Stefania Ruggeri | | |  |
| IV. | Interim data | The modifications were not informed by interim trial data or study outcomes. They were based on ethical and operational considerations. | | |  |
| CONSORT Number and Item | | For each row, if important modifications occurred check “direct impact” and/or “mitigating strategy” and describe the changes in the trial manuscript or supplement. Check “no change” for items that are unaffected in the extenuating circumstance. | | | Manuscript section |
|  |  | No Change | Impact* | Mitigating Strategy** |  |
| 1 | Title and abstract | X |  |  |  |
| 2 | Introduction | X |  |  |  |
| 3 | Methods: Trial Design |  | the Rome school-based component of the AUT-MENU study initiated later than originally planned due to the ethical approval process. | The number of planned meal observations per participant was reduced from 16 to 12 to accommodate students’ attendance at school canteen. | Materials and Methods - Study Design and Setting |
| 4 | Methods: Participants |  | One of the two registered Rome schools (IPSEOA “Tor Carbone” high school) could not be included because an on-site canteen service required for the planned study activities was not available. | The Rome school-based component of the AUT-MENU study was conducted at the participating school (“Giuseppe Garibaldi-Agricultural Technical Institute” high school) with an available on-site canteen service. The upper age limit was extended to 20 years to allow the participation of eligible students attending the school. | Materials and Methods - Study Design and Setting |
| 5 | Methods: Interventions | X |  |  |  |
| 6 | Methods: Outcomes | X |  |  |  |
| 7 | Methods: Sample Size |  | The estimated enrollment reported in the registered trial refers to the overall bicentric study and was not specific to the Rome school-based component of the AUT-MENU study, in which 14 participants were enrolled and 11 completed the study. |  | Materials and Methods – Enrolment and Eligibility Criteria |
| 8-10 | Methods: Randomisation | X |  |  |  |
| 11 | Methods: Blinding | X |  |  |  |
| 12 | Methods: Statistical methods | X |  |  |  |
| 13 | Results: Participant flow | X |  |  |  |
| 14 | Results: Recruitment | X |  |  |  |
| 15 | Results: Baseline data | X |  |  |  |
| 16 | Results: Numbers analysed | X |  |  |  |
| 17 | Results: Outcomes and estimation | X |  |  |  |
| 18 | Results: Ancillary analyses | X |  |  |  |
| 19 | Results: Harms | X |  |  |  |
| 20 | Discussion: Limitations | X |  |  |  |
| 21 | Discussion: Generalisability |  | The Rome school-based component of the AUT-MENU study was conducted at a single high school, which may limit the generalisability of the findings to other school canteen settings. |  | Limitations and Strengths of the Study |
| 23 | Other information: Registration |  | The Rome school-based component of the AUT-MENU study differs from the original ClinicalTrials.gov registration with respect to the eligible age range, participating study sites, study timeline, and meal-assessment schedule. | The manuscript was revised to clarify that the present paper reports data from the Rome school-based component of the AUT-MENU study as a part of the registered bi-centric study and to report the deviations from the original trial registration. The modifications were documented in accordance with the CONSERVE-CONSORT guidelines. | Materials and Methods - Study Design and Setting |
| 24 | Other information: Protocol | X |  |  |  |
| 25 | Other information: Funding | X |  |  |  |
| *Aspects of the trial that are directly affected or changed by the extenuating circumstance and are not under the control of investigators, sponsor or funder.  **Aspects of the trial that are modified by the study investigators, sponsor or funder to respond to the extenuating circumstance or manage the direct impacts on the trial. | | | | | |
